# The Leigh phenotype resulting from *C12orf65* variants

**DOI:** 10.1590/1678-4685-GMB-2020-0177

**Published:** 2020-08-17

**Authors:** Josef Finsterer

**Affiliations:** 1 Krankenanstalt Rudolfstiftung, Vienna, Austria.

With interest we read the article by Perrone *et al.* (2020) about a 2 years old female with Leigh syndrome (LS) due to the homozygous 14bp frameshift deletion in *C12orf65* encoding COXPD7. It was concluded that the mutated protein domain GGQ is responsible for a conservative peptidyl-hydrolase function and that loss of function mutations in this domain play a pathogenetic role for the development of LS (Perrone *et al.*, 2020). We have the following comments and concerns.

We do not agree with the notion that LS is exclusively an early onset disease (Perrone *et al.*, 2020). Though presenting with an onset <2y of age in 80% of the cases (Hong *et al.*, 2020), the remaining portion of LS patients has an onset >2y of age (Hong *et al.*, 2020).

We also do not agree with the statement that “brain changes are almost identical in all patients” with LS (Perrone *et al.*, 2020). Though cerebral lesions are usually symmetric with regard to distribution, different structures may be affected. These include the caudate nucleus, the putamen, the globus pallidus, the thalamus, the brainstem, or the cerebellum (Finsterer, 2008). Though these miscellaneous presentations on imaging do not allow establishing close genotype-phenotype correlations, they represent to some extent the extensive genetic heterogeneity of the syndrome.

We should know if respiratory distress episodes were attributed to the brainstem lesion in the medulla oblongata, to affection of peripheral nerves affecting respiratory muscles, to myopathy of axial including respiratory muscles, or to lactic acidosis.

Though the *C12orf65* deletion was present in the homozygous state and though DNA from the parents was extracted it is not mentioned if either parent carried the mutation in the heterozygous state, as could be expected. Sporadic occurrence of the variant is rather unlikely given the homozygous state.

Missing in the report is if lactate was measured in the cerebro-spinal fluid (CSF) or not. Neither did the patient undergo magnetic resonance spectroscopy (MRS), nor was a spinal tap carried out. Since cerebral lactic acidosis strongly determines the phenotype it is crucial to know if lactate was elevated in the CSF respectively the parenchyma or not.

Since some patients with LS may develop stroke-like episodes (SLEs) (Morin *et al.*, 1999), we should know if the patient ever developed features of a SLE and if any of the cerebral MRIs ever showed a characteristic stroke-like lesion (SLL), the morphological equivalent of a SLE.

Since LS may go along with epilepsy and since epileptiform discharges were recorded on electroencephalography (EEG) (Perrone *et al.*, 2020), we should know if the patient ever developed seizures or not and if treatment with anti-epileptic drugs (AEDs) was ever necessary or not.

Since cerebral MRI at age 2y and 9 months revealed bilaterally symmetric T2-hyperintense lesions in the substantia nigra, we should know if the patient ever developed Parkinsonism or a Parkinson-related disorder. Did the patient ever require anti-Parkinson drugs?

Overall, this interesting case has a number of shortcomings, which should be solved before the case is revaluated and final conclusions are drawn. Patients with LS are not only heterogeneous with regards to the genetic background but also with regard to the phenotype.
