# Supplementary material for: The Leigh phenotype resulting from C12orf65 variants
Source: Genet Mol Biol. 2020 Aug 17;43(3):e20200177. doi: 10.1590/1678-4685-GMB-2020-0177 (PMC7433753; doi:10.1590/1678-4685-GMB-2020-0177)
Supplement: Related texts

# Author's reply to the comment by J. Finsterer

**Authors:** Eduardo Perrone

**Affiliations:** 1 Universidade Federal de São Paulo, Departamento de Genética Médica, São Paulo, SP, Brazil.

We really appreciated the interest and comments on our article (Perrone *et al.*, 2020). Although we agree that different central nervous system structures can be affected in Leigh syndrome, and that there is considerable clinical, genetic, and biochemistry heterogeneity, when we pointed out that lesions were almost identical, we aimed to highlight symmetrical basal ganglia, brain stem, and cerebellum involvement, which is in line with previous descriptions (Finsterer, 2008).

Our neurological and clinical evaluation was limited, considering that the proband was under sedation and on mechanical ventilation. As the patient had brainstem signals in MRI, we believe that respiratory distress was mainly caused by brainstem involvement. However, it is not possible to rule out the influence of lactic acidosis, myopathy, or even peripheral nerve disorders. For your information, the CSF lactate level was 45.22 mg/dL (upper limit, 20 mg/dL). We did not observe signals of stroke-like episodes (SLE) in our patient. Partial complex seizures were observed, partially controlled by carbamazepine, phenobarbital, and clobazam. MRI also revealed substantia nigra lesions, but we were not able to identify any Parkinsonism signs, considering all the above-mentioned limitations. Spectroscopy MRI would be informative, but it was not performed.

Regarding the genetic aspects, the consent was signed only by the mother, and we had access only to the proband's biological samples. Due to ethical and legal issues we could not contact the proband's father. However, parental consanguinity strongly suggests that the proband inherited identical pathogenic variants from both parents.

## References

[B5] Finsterer J (2008). Leigh and Leigh-like syndrome in children and
adults. Pediatr Neurol.

[B6] Perrone E, Cavole TR, Oliveira MG, Virmond LDA, Silva MFB, Soares MFF, Iglesias SBO, Falconi A, Silva JS, Nakano V (2020). Leigh syndrome in apatient with a novel C12orf65 pathogenic
variant: Case report and literature review. Genet Mol Biol.
